# Three-Dimensional Quantitative Magnetic Resonance Imaging Cartilage Evaluation of the Hand Joints of Systemic Sclerosis Patients: A Novel Insight on Hand Osteoarthritis Pathogenesis—Preliminary Report

**DOI:** 10.3390/jcm12237247

**Published:** 2023-11-23

**Authors:** Michał Waszczykowski, Michał Podgórski, Jarosław Fabiś, Arleta Waszczykowska

**Affiliations:** 1 Department of Arthroscopy, Minimally Invasive Surgery and Sports Traumatology, Medical University of Lodz, Kosciuszki 4, 90-419 Lodz, Poland; fabis@onet.eu; 2 3rd Radiology Department, WAM Hospital, Medical University of Lodz, Zeromskiego 113, 90-549 Lodz, Poland; podgorskimt@gmail.com; 3 Department of Ophthalmology, Medical University of Lodz, Kopcinskiego 22, 90-153 Lodz, Poland; arletawaszczykowska@wp.pl

**Keywords:** systemic sclerosis, hand, cartilage, magnetic resonance imaging, arthritis, osteoarthritis, acro-osteolysis

## Abstract

Background. Osteoarthritis of the hand joints in systemic sclerosis (SSc) patients might be an independent manifestation leading to limitation of upper extremity function. There is no publication quantitatively assessing the thickness of articular cartilage within the hand joints of SSc patients by MRI. The purpose of our study was to quantify the condition and thickness of hand joints cartilage with three-dimensional quantitative MRI (3D q-MRI). Methods. The study was conducted in twenty people: ten patients with SSc and ten healthy individuals. All participants were examined with the 3D q-MRI with 3T scanner. The cartilage thickness of proximal (PIP) and distal interphalangeal (DIP) joints as well as metacarpophalangeal joints was measured. Results. There was no significant difference in cartilage thickness between both groups. However, the joint cartilage was thinner in fingers with acro-osteolysis. In PIP joint of the fingers with acro-osteolysis, the mean cartilage thickness was 0.5 mm (*p* = 0.0043) and 0.4 mm (*p* = 0.0034) in DIP joints. Conclusions. Quantitative MRI analysis of the joints of the hands of SSc patients does not indicate changes in thickness of the articular cartilage. A significant reduction in the articular cartilage thickness of the fingers with acro-osteolysis indicates the potential of an ischemic basis of articular cartilage destruction in SSc patients.

## 1. Introduction

Systemic sclerosis (SSc) is an autoimmune connective tissue disease in which there is marked deposition and accumulation of collagen and severe disruption of the vascular system of the skin and internal organs [1,2,3]. Increased synthesis of extracellular matrix proteins and fibroblast proliferation lead to damage to the vascular bed and impaired function of numerous systems [4]. The most common pathological changes involve the skin, pulmonary system, gastrointestinal tract, musculoskeletal system, and urinary tract. In clinical practice, the examination and diagnosis among these patients is based primarily on the evaluation of the condition of the skin and involved internal organs. However, these patients are also accompanied by pathological changes in the musculoskeletal system, which particularly affect the hands [5,6]. Progressive skin thickening and adhesions lead to joint contractures and progressive limitation of upper extremity function [5,6,7]. However, these changes are not the only ones responsible for pain, reduced range of motion in the hand joints, and impaired function. Recent studies indicate that clinical symptoms of peripheral hand osteoarthritis may be present in more than 61% of patients [8,9]. Moreover, these studies indicate that arthritis of the hand joints in these patients may be an independent manifestation of osteoarthrosis, rather than an RA overlap syndrome as previously thought [10,11,12]. Imaging diagnosis in these patients to assess the severity of osteoarthrosis lesions in the hand joints is based primarily on classic radiography and ultrasonography [5]. A small number of publications show the value of nuclear magnetic resonance imaging in the evaluation of hand joint osteoarthritis (OA) in patients with SSc [5,13,14,15]. They present a qualitative assessment of pathological changes in hand joint OA in SSc patients in the form of erosions, bone and marrow oedema, synovitis, joint effusion, and tendovaginitis [13,14,15]. However, to date, no publication has appeared quantitatively assessing the thickness of articular cartilage within the hand joints of SSc patients by nuclear magnetic resonance imaging.

The purpose of our study, therefore, was to evaluate and quantify the condition and thickness of hand joint cartilage in SSc patients with a three-dimensional quantitative MRI (3D q-MRI) 3T scanner. We also wanted to determine what the potential mechanism of destruction of the cartilage of the hand joints in these patients might be.

## 2. Materials and Methods

### 2.1. Study Design: Study and Control Group

The prospective two-arm observational study was conducted in twenty people: ten patients who met the criteria for diagnosing systemic sclerosis (SSc) according to the American College of Rheumatology/European League Against Rheumatism (ACR/EULAR) 2013 Classification Criteria [16] and ten individuals who were the control group of healthy people (HC). The other eligibility criteria for SSc patients were as follows: adults (>18 years old), pain, oedema, contracture or limitation of range of motion of at least one joint of the hand. Further characteristics of the studied groups are presented in Table 1. The main exclusion criteria were as follows: history indicating the presence of other autoimmune diseases (rheumatoid arthritis, ankylosing spondylitis) or clinical picture of overlap syndrome, cancer and dysfunction of the upper limbs caused by past injury. All patients were consecutively enrolled to this study from the outpatient and inpatient clinic of the Department of Dermatology and Venerology of the University of Lodz (Poland) and were referred to the outpatient orthopedics clinic and radiological outpatient department. 

The control group consisted of persons corresponding to age and gender distribution for the study group, in whom no clinical and radiological symptoms of degenerative disease nor features of the SSc and arthritis of the hands were found and no other criteria were found to exclude them from the study.

All the patients and control group members agreed by a written informed consent to participate in the study. The study was conducted in accordance with the Declaration of Helsinki. Ethical approval for the study was obtained from the Bioethics Committee of the Medical University of Lodz (consent number: RNN/332/06/KB).

### 2.2. Clinical Examination

**Figure 1:**
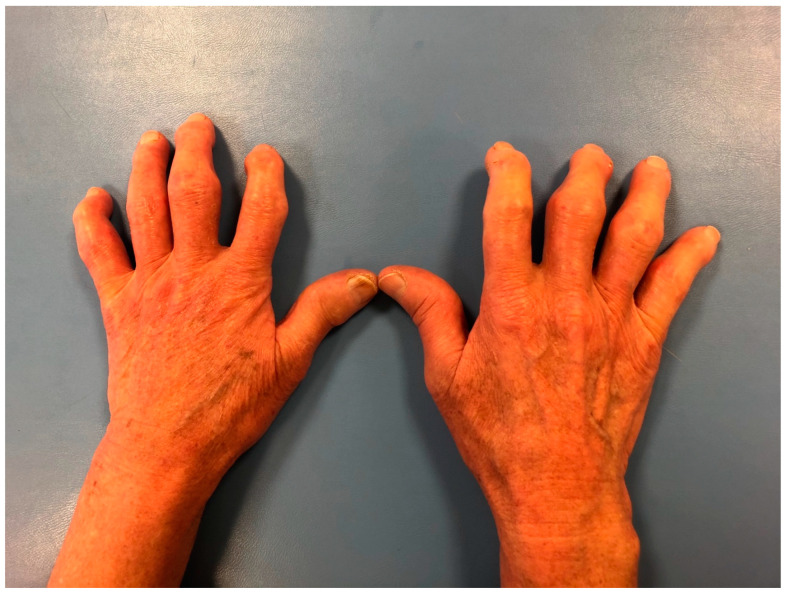
Clinical manifestations of pathological changes in the hands in SSc patient: thickening of the skin and subcutaneous tissue; claw-type deformity of the fingers: a limited extension in proximal and distal interphalangeal joints (PIP, DIP); a hyperextension in metacarpophalangeal joints (MCP); oedema and deformities of proximal and distal interphalangeal joints (PIP, DIP).

All patients underwent a clinical examination with the assessment of duration of the disease and Reynaud’s phenomenon, skin status on the hands and feet with modified Rodnan skin score, as well as ulceration of the fingers and fingertip (Figure 1). The presence of accompanying pathological changes in the lungs (pulmonary fibrosis), cardiovascular involvement, gastrointestinal manifestation, hematological involvement, and renal abnormalities were also noted (Table 1).

The patients with limited cutaneous systemic sclerosis (lcSSc) were treated with vasodilating drugs (calcium channel blockers, angiotensin receptor antagonists or pentoxifylline) and vitamin E. The diffuse cutaneous systemic sclerosis (dcSSc) patients were treated with immunosuppressive therapy (Metypred 8 mg, low doses of corticosteroids—Prednisone 0.5 mg/kg bw/day) alone or in combination with cytostatic therapy (cyclophosphamide 1.5 mg/kg bw/day), pentoxifylline (Polfilin), vitamin E, mucolytic agents (Acetylcysteine), Omeprazole (Bioprazole), Dextran infusion.

### 2.3. Radiological (X-ray) Evaluation

**Figure 2:**
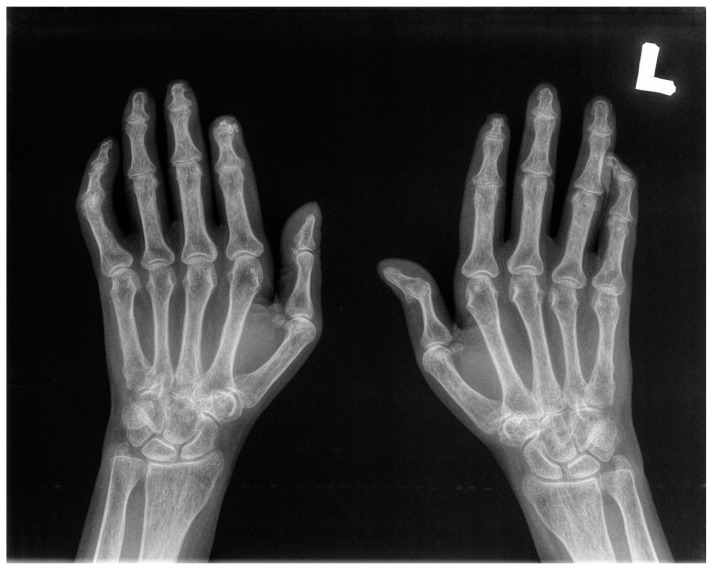
Radiological manifestations of pathological changes in the hands in SSc patients’: Resorption of bilateral 2nd distal phalanxes and 1st phalanx of the right hand (acro-osteolysis); joint space narrowing of PIP and DIP joints; erosions and juxta-articular osteopenia; calcinosis of the 2nd fingertip.

The X-ray examination of both hands was conducted in all participants (Figure 2). 

### 2.4. Magnetic Resonance Imaging

**Figure 3:**
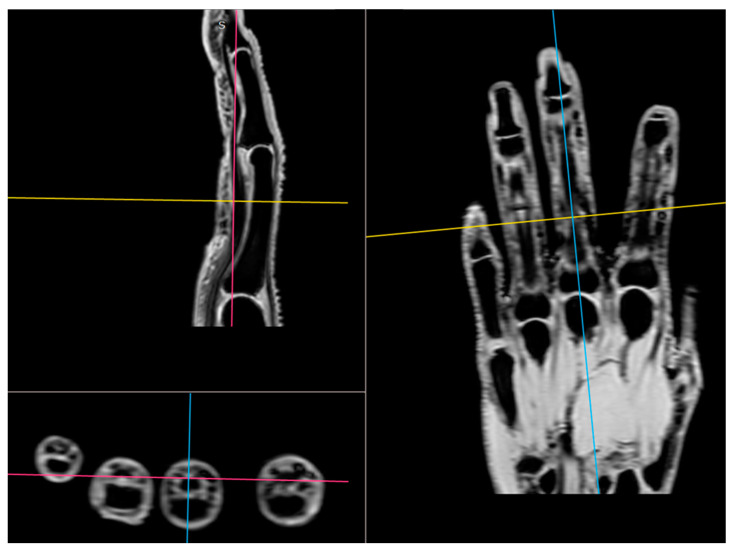
The application of a 3D sequence of MRI to obtain the true sagittal cross-section of each finger.

**Figure 4:**
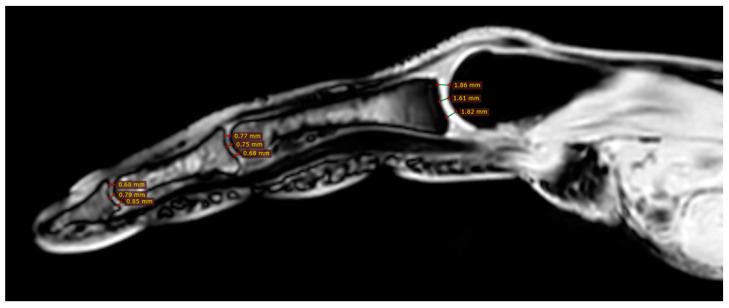
Three measurements of cartilage thickness in each finger joint (in volar 1/3, dorsal 1/3, and middle 1/3)—finger without acro-osteolysis.

**Figure 5:**
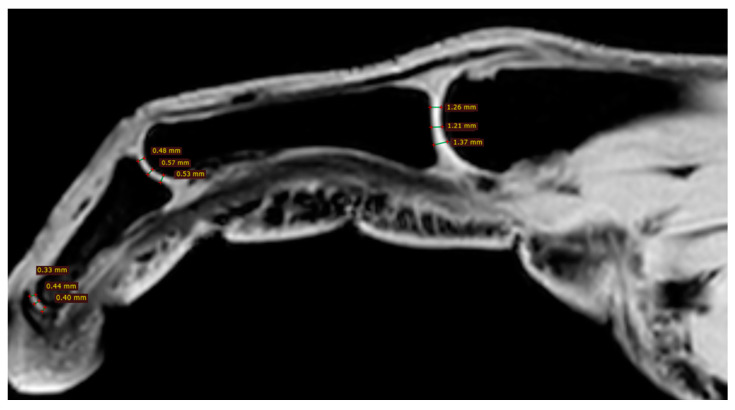
Three measurements of cartilage thickness in each finger joint (in volar 1/3, dorsal 1/3, and middle 1/3)—finger with acro-osteolysis.

All participants were examined with the three-dimensional quantitative MRI (3D q-MRI). The Philips 3T scanner was used with the coil for small parts. After obtaining structural T1 and T2 scans, the WATS 3D sequence was performed. The WATS sequence is dedicated to the cartilage evaluation and highlights the subchondral cortical layer. Due to the application of a 3D sequence, we were able to obtain the true sagittal cross-section of each finger (Figure 3). This was vital because, in some patients, contractions did not allow for anatomical position of fingers during examination. After adjusting the planes, we measured the distance between two cortical layers for each finger joint in its middle transverse dimension, obtaining three measurements (in volar 1/3, dorsal 1/3, and middle 1/3) and calculating a mean (Figure 4 and Figure 5). The distal phalanx length (DPL), the cartilage thickness of distal interphalangeal joints (DIPJ), proximal interphalangeal joints (PIPJ), interphalangeal joint (IPJ), and metacarpophalangeal joints (MCPJ) in all participants were measured. 

### 2.5. Statistical Analysis

In statistical analysis the Statistica 13 software [TIBCO Software Inc., Palo Alto, CA, USA, 2017] was used. 

First, the Shapiro–Wilk test was used to evaluate for normality of continuous data distribution. Distribution was other than normal; thus, to compare morphological parameters, the following tests were used:

- The Manny–Whitney test to compare cartilage thickness and distal phalanx length between individual fingers and joints of patients from two groups.
- The Friedman ANOVA with posthoc test to evaluate differences in cartilage thickness and distal phalanx length within fingers of the same patients from both groups. This test was also used to compare the difference in cartilage thickness between the DIPJ, PIPJ, and MCPJ of fingers with acro-osteolysis.

A *p*-value of less than 0.05 was considered significant, with Bonferroni correction for multiple testing. Results are presented as mean and standard deviation unless otherwise stated.

## 3. Results

Ten SSc patients with mean age 62.6 (range 57 to 71) were included in the study. Nine of them were female. Seven patients suffered from a limited form of SSc (lcSSc). Six patients presented gastrointestinal manifestations. Four patients demonstrated pulmonary fibrosis. Six patients showed cardiac abnormalities. One of them presented renal pathological involvement. Detailed characteristics of the patients are provided in Table 1. All the patients had clinical signs of hands arthralgia. On clinical examination, we found signs of acro-osteolysis confirmed by X-ray in four patients. Three patients manifested the symptoms of digital ulcers; two of them had signs of calcinosis of distal phalanx (Table 1). The X-ray examinations of both hands were conducted in all ten patients. They revealed osteolysis of proximal and distal phalanxes (acro-osteolysis), joint space narrowing of DIPJs and PIPJs, erosions and juxa-articular osteopenia, as well as calcinosis of the fingertips (Figure 2). Ten healthy volunteers were included in the study as a control group. There were no statistically significant differences between both groups in terms of age and gender. 

### Cartilage Thickness and Distal Phalanx Length in MRI

In general comparison, there was no significant difference in all evaluated parameters. The same was for the comparison of parameters between fingers of the same hand (DPL *p* = 0.2925, DIPJ *p* = 0.1800, PIPJ *p* = 0.0898; MCPJ *p* = 0.1893; pulled data from both groups, Table 2) and for the comparison of cartilage thickness within joints of fingers 2–5. (finger 2. *p* = 0.6958; finger 3. *p* = 0.6857, finger 4. *p* = 0.2646; finger 5. *p* = 0.5122; pulled data from both groups, Table 2). In finger 1, the cartilage of MCPJ was significantly thicker than that of IPJ (*p* = 0.0005).

Despite the insignificant results above, we noticed that joint cartilage is thinner in fingers with acro-osteolysis (Figure 4 and Figure 5). In the PIP joint of fingers with acro-osteolysis, the mean cartilage thickness was 0.5 mm (*p* = 0.0043). In the DIP joint of fingers with acro-osteolysis, the mean cartilage thickness was 0.4 mm (*p* = 0.0034). In the MCP joint, we did not find a statistically significant difference between the study and control group (*p* = 0.8554). In Table 3, there is a comparison of parameters between these fingers and all the rest from both groups.

## 4. Discussion

To the best of our knowledge and the current literature, this study is the first to quantitatively analyze the cartilage status of the hand joints of SSc patients via MRI. In addition, this is the first publication to analyze the cartilage status of the hand joints of these patients via MRI in general. To date, available studies have shown joint and periarticular changes on MRI imaging in a qualitative manner, but without evaluating the articular cartilage itself [5,13,14,15,17,18,19]. It is believed that symptoms of hand arthritis in patients with SSc, manifested by joint pain and swelling, may be present in more than 60% of patients [8,9]. These symptoms, together with progressive thickening of the skin and adhesions in the finger flexor tendons, lead to permanent upper limb dysfunction [4,7,20]. The pathogenesis of arthritis in patients with SSc still seems controversial [8]. According to some authors, an overlap syndrome with RA is responsible for it [21,22]. This may be indicated by a similar histopathological picture of synovitis as in RA, which initiates the process of joint destruction [23]. These hypotheses may be further supported by the observation of radiological images from US and MRI, in which synovitis, tendosynovitis, joint effusion, and erosions were found [14,17,18]. In contrast, others believe that the arthritis symptoms in these patients are an independent manifestation of hand osteoarthritis, independent of RA [8]. 

The development of diagnostic imaging in recent years has provided a more accurate understanding of the causes and pathomechanism of many diseases. MRI has become the standard in imaging lesions and pathologies within the musculoskeletal system. SSc is also gaining a number of new applications, increasing the knowledge of this disease and increasing the diagnosis and effectiveness of treatment. There are publications in the available literature that have analyzed MRI images of pathological lesions in the hands of patients with SSc [5,13,14,15,17,18,19]. To date, however, these have only been qualitative analyses and have focused primarily on evaluating the synovium of the hand joints and tendon sheaths, bone marrow edema, and erosions within the joints [5,13,14,15,17,18,19]. Chitale et al., in their study, demonstrated greater sensitivity of MRI than US in the evaluation and detection of synovitis, bone marrow oedema, and erosion in patients with SSc [19]. They used the semiquantitative RAMARIS scale to evaluate MRI. These observations were confirmed several years later by Abdel-Magied et al. [15]. In a group of 16 SSc patients with arthralgia in the hands but without clinical signs of arthritis, they confirmed the greater sensitivity of MRI in comparison with US in the evaluation and detection of arthritic lesions. Moreover, they showed that these lesions occur in patients who do not manifest clinical signs of arthritis [15]. These studies have made a clear and important contribution to the understanding of the pathology and changes taking place within the joint and periarticular structures, pointing to arthritis as one of the potential causes of hand dysfunction and SSc disease picture [13,14,15,17,18,19]. We were the first in the available literature to analyze the condition of the articular cartilage of the hands in these patients and quantify it. All patients included in this study had clinical signs of hand arthralgia with a marked effect on hand function impairment [20]. All participants (study and control group) were examined with the three-dimensional quantitative MRI (3D q-MRI). The Philips 3T scanner was used with the coil for small parts. We evaluated the length of the distal phalanx of each finger, the articular cartilage thickness of the MCP joints of fingers 1–5, the PIP joints, the DIP joints of fingers 2–5, and the IP of finger 1. In an overall analysis of the results, we found no statistically significant differences in both the cartilage thickness of the joints studied and the length of the distal phalanx between the two groups of participants. As we mentioned earlier, our study is the first to evaluate the condition and cartilage thickness of hand joints in patients with SSc. Therefore, we are unable to relate these results to other such measurements and studies. The lack of statistically significant differences in cartilage thickness measurements between patients with SSc and the healthy population may be due to the fact that despite inflammatory changes taking place in and around the joints, there is no noticeable destruction of joint cartilage. This may therefore indicate that the manifestation of arthralgia-like lesions is primarily associated with periarticular changes, tendosynovitis, and thickening of the skin around the joints of the hands. The previously cited studies that used MRI to qualitatively evaluate joint and periarticular lesions noted that in addition to synovitis and tendosynovitis, bone marrow oedema and erosions were also observed [14,15,17,18,19]. In addition, Low et al. and also Rutka et al. indicate that in MRI images, qualitative intra-articular and periarticular lesions of synovitis, tendinitis, bone marrow oedema, and erosions may be present in more than 50% of patients, but do not correlate with the clinical picture [5,14]. At the same time, the results of our study indicate an important observation that has not been reported so far. We noticed that joint cartilage is thinner in fingers with acro-osteolysis. In the PIP joint of fingers with acro-osteolysis, the mean cartilage thickness was 0.5 mm (*p* = 0.0043). In the DIP joint of fingers with acro-osteolysis, the mean cartilage thickness was 0.4 mm (*p* = 0.0034). Acro-osteolysis is a radiographic sign of bone resorption of the distal phalanges of the distal phalanges of the fingers also seen in patients with SSc [24,25,26]. Sakata et al., in their radiological analysis of the hands of SSc patients, showed acro-osteolysis in 16% of patients [27]. On the other hand, Erre et al. found in an X-ray analysis of SSc patients that the sign of acro-osteolysis was present in 17% of the study population [11]. On the other hand, Babulal Vadher et al. found in their study that in MRI analysis, phalangeal acro-osteolysis was present in 28% of patients in the study population [18]. Avouac et al. as well as Koutaissoff et al. and Johnstone et al. indicate in their studies that there is a positive correlation between the occurrence of acro-osteolysis sign and its severity, and finger ischemia (RP) [24,25,26]. Sakata et al., on the other hand, found a positive correlation between the incidence of acro-osteolysis and the duration of the disease, accompanying respiratory changes and the form of dsSSc [27]. The lesions also correlated positively with digital tip ulcers and digital pitting scars, i.e., pathological changes of a chronic ischemic nature [27]. Similar observations were made by Johnstone et al. They found that the frequency of phalangeal acro-osteolysis correlates positively with the duration of ischemia in the fingers and the duration of the disease [24]. In contrast, Avouc and colleagues found in their prospective study that factors predictive of future acro-osteolysis were digital ulcers and calcinosis at baseline [6]. An interesting observation was made by Akbayrak et al. [13]. In their study, they conducted an analysis of pathological OA-like lesions in the hand joints of SSc patients using low-filed MRI. They noted bone marrow oedema and erosions in the lunar bone, which may indicate early osteonecrosis changes in this bone. The avascular necrosis (AVN) of the lunar bone and the observed osteoarthritic changes indicate that the background of these changes may not only be autoimmune, but also ischemic. Previous publications indicate a correlation between the severity of Reynaud’s phenomenon and the symptoms of AVN of the lunar bone [28,29]. Our findings may indicate a hitherto undescribed new mechanism of hand joint arthropathy in patients with SSc. Destruction of articular cartilage in the fingers with signs of acro-osteolysis may indicate an ischemic mechanism for this process. Avouac et al., in their study, found a positive correlation between the severity of Reynaud’s phenomenon and the development of erosive arthritis in SSc [6]. This may support the hypothesis that the development of OA-like lesions in these patients may be influenced by chronic ischemia. Siao-Pin et al., in their meta-analysis, further point to hypoxia as a key phenomenon that may be largely responsible for the development of acro-osteolysis lesions in SSc patients [30]. Hypoxia may enhance osteoclastogenesis and increase vascular endothelial growth factor (VEGF) activity, which may contribute to bone resorption and acro-osteolysis, as in other manifestations of the disease [30,31]. Perhaps it is this hypoxic mechanism and impaired angiogenesis that are also responsible for the destruction of articular cartilage and the development of osteoarthrosis in the distal interphalangeal joints of the SSc patients studied. Almeida et al. proved in their study that increased angiostatin levels in SSc patients correlate positively with osteoarthrosis symptoms in these patients [32]. 

Our study unfortunately has some limitations. The first is the small size of the study group. As we indicated earlier, our analysis is a preliminary report. The results of our study need to be confirmed on a larger population in order to formulate more objective conclusions. Another weak point of our analysis is the lack of correlation of the results of articular cartilage thickness with parameters of angiogenesis processes, which would increase the value and importance of our conclusions. Undoubtedly, our observations require further studies of the quantitative assessment of articular cartilage in SSc patients, the mechanisms and causes of its degradation, and their interrelationships with the processes of angiogenesis and bone resorption.

## 5. Conclusions

Quantitative MRI analysis of the joints of the hands of SSc patients with arthralgia symptoms does not indicate changes in the structure and thickness of the articular cartilage. However, a significant reduction in the thickness of the articular cartilage of the fingers affected by acro-osteolysis indicates that the process of articular cartilage destruction in SSc patients may have an ischemic basis.

## Figures and Tables

**Table 1:** Baseline characteristics of study group.

Clinical Characteristics	Study Group (n = 10)
Age: mean, range (±SD)	62.8, 57–71 (5.7)
Women	9 (90%)
Skin score (mRSS): mean, (±SD)	13.1 (6.4)
Type of SSc	
lcSSc	7
dcSSc	3
Duration of the SSc, mean, range, (±SD)	10.3, 6–22 (4.7)
Gastrointestinal manifestation	6 (60%)
Pulmonary fibrosis	4 (40%)
Cardiac involvement	6 (60%)
Renal abnormalities	1 (10%)
Hematological involvement	2 (20%)
Acro-osteolysis (*)	4 (11)
Digital ulcers	3 (30%)
Calcinosis	2 (20%)

The outcomes are shown as a number and precent. Values are the mean, range, and ±SD. SSc—systemic sclerosis; lcSSc—limited cutaneous systemic sclerosis; dcSSc—diffuse cutaneous systemic sclerosis; mRSS—modified Rodnan skin score. * Number of fingers with acro-osteolysis.

**Table 2:** A between-group comparison of distal phalanx length and cartilage thickness in each finger joint.

Finger	Parameter	Scleroderma	Control	*p*-Value
1	DPL	16.9 (5.5)	19.0 (1.2)	0.4309
	IPJct	0.8 (0.2)	0.8 (0.1)	0.7929
	MCPJct	1.1 (0.1)	1.1 (0.1)	0.6365
2	DPL	12.7 (5.2)	15.8 (1.4)	0.4008
	DIPJct	0.7 (0.3)	0.8 (0.1)	0.4948
	PIPJct	0.9 (0.5)	0.9 (0.2)	0.3720
	MCPJct	1.4 (0.3)	1.5 (0.3)	0.5635
3	DPL	14.0 (4.6)	15.1 (1.0)	0.8748
	DIPJct	0.7 (0.3)	0.7 (0.2)	0.7132
	PIPJct	0.8 (0.2)	0.8 (0.1)	0.7132
	MCPJct	1.2 (0.2)	1.1 (0.1)	0.9581
4	DPL	16.4 (1.5)	15.3 (0.9)	0.3720
	DIPJct	0.9 (0.2)	0.9 (0.1)	0.9581
	PIPJct	0.9 (0.2)	0.8 (0.4)	1.0000
	MCPJct	1.1 (0.3)	1.1 (0.2)	0.9581
5	DPL	15.1 (1.5)	15.0 (0.8)	0.7527
	DIPJct	0.8 (0.2)	0.8 (0.2)	0.9164
	PIPJct	0.9 (0.2)	0.8 (0.1)	0.4309
	MCPJct	1.2 (0.2)	1.0 (0.2)	0.0929

Abbreviations: DPL—distal phalanx length; IPJ—interphalangeal joint; DIPJ—distal interphalangeal joint; PIPJ—proximal interphalangeal joint; MCPJ—metacarpophalangeal joint; ct—cartilage thickness. The significant *p* level according to Bonferroni correction is 0.002. All values are given in millimeters.

**Table 3:** Comparison of distal phalanx length and cartilage thickness between fingers with acro-osteolysis and all the rest.

Parameter	Acro-Osteolysis (11 Fingers)	Normal (89 Fingers)	*p*-Value
DPL	4.9 (1.77)	16.2 (2.33)	0.0044
DIPJct	0.4 (0.04)	0.8 (0.2)	0.0034
PIPJct	0.5 (0.04)	0.8 (0.2)	0.0043
MCPJct	1.3 (0.2)	1.2 (0.2)	0.8554

Abbreviations: DPL—distal phalanx length; IPJ—interphalangeal joint; DIPJ—distal interphalangeal joint; PIPJ—proximal interphalangeal joint; MCPJ—metacarpophalangeal joint; ct—cartilage thickness. The significant *p* level according to Bonferroni correction is 0.002. All values are given in millimeters.

## Data Availability

The data used to support the findings of this study will be available at the request of the corresponding author.
